# Tetra­aqua­bis­(pyridine-κ*N*)nickel(II) dinitrate

**DOI:** 10.1107/S1600536810021653

**Published:** 2010-06-16

**Authors:** Mario Wriedt, Inke Jess, Christian Näther

**Affiliations:** a Institut für Anorganische Chemie, Christian-Albrechts-Universität Kiel, Max-Eyth-Strasse 2, 24098 Kiel, Germany

## Abstract

In the title compound, [Ni(C_5_H_5_N)_2_(H_2_O)_4_](NO_3_)_2_, the Ni^II^ ion is coordinated by two *N*-bonded pyridine ligands and four water mol­ecules in an octa­hedral coordination mode. The asymmetric unit consists of one Ni^II^ ion located on an inversion center, as well as one pyridine ligand, one nitrate anion and two water mol­ecules in general positions. In the crystal structure, the discrete complex cations and nitrate anions are connected by O—H⋯O and C—H⋯O hydrogen bonds.

## Related literature

For general background to thermal decomposition reactions as an alternative tool for the discovery and preparation of new ligand-deficient coordination polymers with defined magnetic properties, see: Wriedt & Näther (2009*a* ▶,*b* ▶); Wriedt *et al.* (2009*a* ▶,*b* ▶). For a related structure, see: Halut-Desportes (1981 ▶).
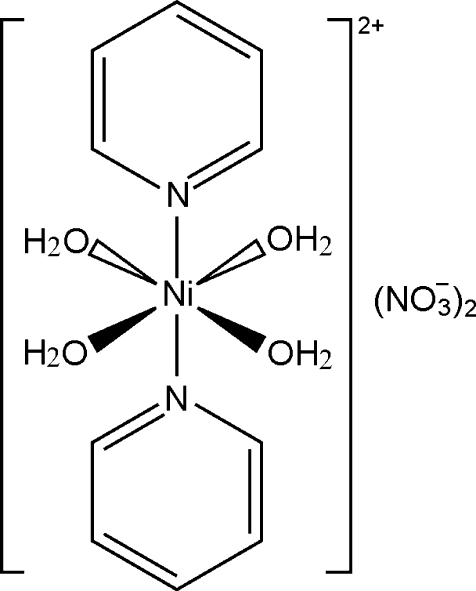


## Experimental

### 

#### Crystal data

[Ni(C_5_H_5_N)_2_(H_2_O)_4_](NO_3_)_2_
*M* *_r_* = 412.99
Monoclinic, 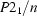

*a* = 7.3245 (4) Å
*b* = 11.3179 (6) Å
*c* = 10.9347 (5) Å
β = 96.436 (4)°
*V* = 900.75 (8) Å^3^
*Z* = 2
Mo *K*α radiation
μ = 1.13 mm^−1^
*T* = 293 K
0.28 × 0.16 × 0.07 mm

#### Data collection

Stoe IPDS-2 diffractometer
Absorption correction: numerical (*X-SHAPE* and *X-RED32*; Stoe & Cie, 2002 ▶) *T*_min_ = 0.801, *T*_max_ = 0.927
12828 measured reflections
2427 independent reflections
2087 reflections with *I* > 2σ(*I*)
*R*_int_ = 0.040

#### Refinement

*R*[*F* ^2^ > 2σ(*F* ^2^)] = 0.049
*wR*(*F* ^2^) = 0.129
*S* = 1.15
2427 reflections
115 parameters
H-atom parameters constrained
Δρ_max_ = 0.32 e Å^−3^
Δρ_min_ = −0.47 e Å^−3^

### 

Data collection: *X-AREA* (Stoe & Cie, 2002 ▶); cell refinement: *X-AREA*; data reduction: *X-AREA*; program(s) used to solve structure: *SHELXS97* (Sheldrick, 2008 ▶); program(s) used to refine structure: *SHELXL97* (Sheldrick, 2008 ▶); molecular graphics: *SHELXTL* (Sheldrick, 2008 ▶); software used to prepare material for publication: *SHELXTL*.

## Supplementary Material

Crystal structure: contains datablocks I, global. DOI: 10.1107/S1600536810021653/hy2315sup1.cif

Structure factors: contains datablocks I. DOI: 10.1107/S1600536810021653/hy2315Isup2.hkl

Additional supplementary materials:  crystallographic information; 3D view; checkCIF report

## Figures and Tables

**Table 1:** Selected bond lengths (Å)

Ni1—O4	2.113 (2)
Ni1—O5	2.128 (2)
Ni1—N1	2.140 (2)

**Table 2:** Hydrogen-bond geometry (Å, °)

*D*—H⋯*A*	*D*—H	H⋯*A*	*D*⋯*A*	*D*—H⋯*A*
O4—H1*O*4⋯O2^i^	0.82	2.39	3.209 (4)	174
O4—H2*O*4⋯O1^ii^	0.82	2.26	3.077 (4)	179
O4—H3*O*4⋯O1	0.82	2.32	3.087 (3)	157
O5—H1*O*5⋯O3^iii^	0.82	2.28	3.091 (4)	169
O5—H2*O*5⋯O1	0.82	2.43	3.191 (4)	155
C2—H2⋯O1^iv^	0.93	2.50	3.310 (4)	145
C4—H4⋯O2^v^	0.93	2.54	3.461 (4)	170

Symmetry codes: (i) 
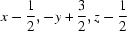
; (ii) 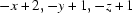; (iii) 
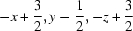
; (iv) 
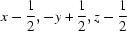
; (v) 
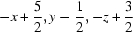
.

**Fig. 1.**
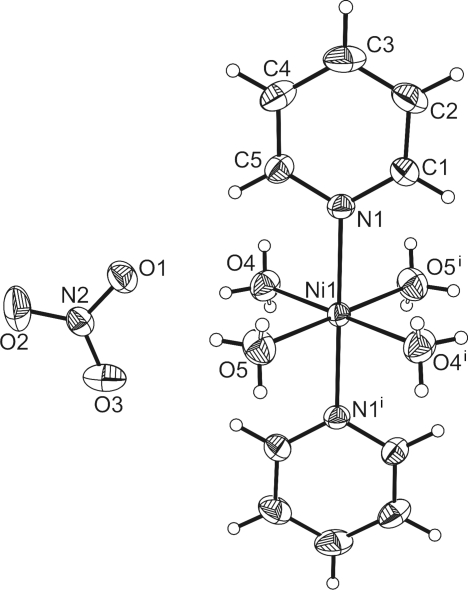
The structure of the title compound with displacement ellipsoids drawn at the 30% probability level. Disordering of the H atoms is shown with full and open bonds. [Symmetry code: (i) -x+1, -y+1, -z+1.]

**Fig. 2.**
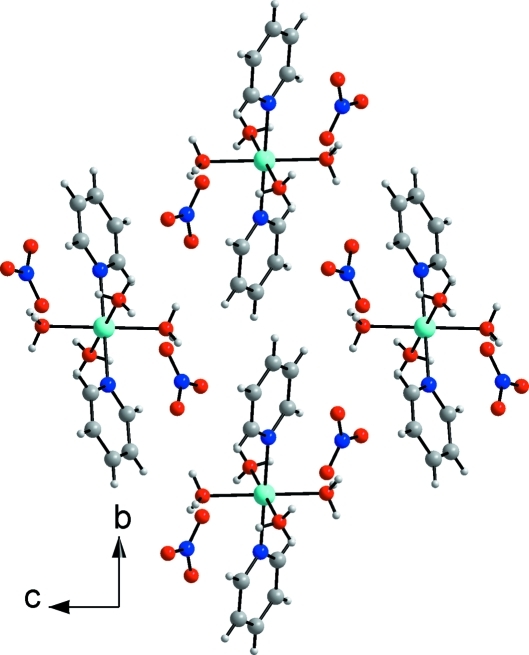
Packing arrangement of the title compound with view along the a axis.

## supplementary crystallographic information

### Comment

Recently, we have shown that thermal decomposition reactions are an elegant route for the discovery and preparation of new ligand-deficient coordination polymers with defined magnetic properties (Wriedt & Näther, 2009a,b; Wriedt *et al.*, 2009a,b). In our ongoing investigation on the synthesis, structures and properties of such compounds based on paramagnetic transition metal pseudo-halides and N-donor ligands, we have reacted nickel(II) dinitrate hexahydrate, sodium dicyanamide and pyridine. In this reaction single crystals of the title compound were grown.

The title compound (Fig. 1) represents a discrete complex cation, in which the Ni^II^ atom, lying on an inversion center, is coordinated by two pyridine ligands and four water molecules in an octahedral coordination mode. The nitrate anions are not coordinated to the metal atoms (Fig. 2). The NiN_2_O_4_ octahedron is slightly distorted with Ni—N_pyridine_ distances of 2.140 (2) Å and Ni—O_water_ distances of 2.113 (2) and 2.128 (2) Å (Table 1). The angles arround the metal atoms range between 85.71 (10) to 94.29 (10) and 180°. A similar coordination is found in a related structure (Halut-Desportes, 1981). The opposite pyridyl rings are coplanar due to symmetry. The shortest intermolecular Ni···Ni distance amounts to 7.3245 (4) Å.

### Experimental

Ni(NO_3_)_2_.6H_2_O (72.7 mg, 0.25 mmol), sodium dicyanamide (44.5 mg, 0.5 mmol) and pyridine (0.5 ml) obtained from Alfa Aesar were reacted in a closed test-tube at 120°C for 3 d. On cooling light green block-shaped single crystals of the title compound were grown in a mixture with unknown phases.

### Refinement

All H atoms were located in a difference Fourier map. H atoms bound to C atoms were positioned geometrically and refined as riding atoms, with C—H = 0.93 Å and with *U*_iso_(H) = 1.2*U*_eq_(C). The water H atoms were disordered over three positions for each water molecule and were refined as riding, with O—H = 0.82 Å and *U*_iso_(H) = 1.5*U*_eq_(O), using a split model with SOF = 0.6667 for each H atom.

### Crystal data

**Table:**

[Ni(C_5_H_5_N)_2_(H_2_O)_4_](NO_3_)_2_	*F*(000) = 428
*M**_r_* = 412.99	*D*_x_ = 1.523 Mg m^−^^3^
Monoclinic, *P*2_1_/*n*	Mo *K*α radiation, λ = 0.71073 Å
Hall symbol: -P 2yn	Cell parameters from 12828 reflections
*a* = 7.3245 (4) Å	θ = 2.6–29.2°
*b* = 11.3179 (6) Å	µ = 1.13 mm^−^^1^
*c* = 10.9347 (5) Å	*T* = 293 K
β = 96.436 (4)°	Block, light green
*V* = 900.75 (8) Å^3^	0.28 × 0.16 × 0.07 mm
*Z* = 2	

### Data collection

**Table:**

Stoe IPDS-2 diffractometer	2427 independent reflections
Radiation source: fine-focus sealed tube	2087 reflections with *I* > 2σ(*I*)
graphite	*R*_int_ = 0.040
ω scans	θ_max_ = 29.2°, θ_min_ = 2.6°
Absorption correction: numerical (*X-SHAPE* and *X-RED32*; Stoe & Cie, 2002)	*h* = −10→9
*T*_min_ = 0.801, *T*_max_ = 0.927	*k* = −15→15
12828 measured reflections	*l* = −15→14

### Refinement

**Table:**

Refinement on *F*^2^	Primary atom site location: structure-invariant direct methods
Least-squares matrix: full	Secondary atom site location: difference Fourier map
*R*[*F*^2^ > 2σ(*F*^2^)] = 0.049	Hydrogen site location: inferred from neighbouring sites
*wR*(*F*^2^) = 0.129	H-atom parameters constrained
*S* = 1.15	*w* = 1/[σ^2^(*F*_o_^2^) + (0.0554*P*)^2^ + 0.6589*P*] where *P* = (*F*_o_^2^ + 2*F*_c_^2^)/3
2427 reflections	(Δ/σ)_max_ < 0.001
115 parameters	Δρ_max_ = 0.32 e Å^−^^3^
0 restraints	Δρ_min_ = −0.46 e Å^−^^3^

### Fractional atomic coordinates and isotropic or equivalent isotropic displacement parameters (Å^2^)

**Table:**

	*x*	*y*	*z*	*U*_iso_*/*U*_eq_	Occ. (<1)
Ni1	0.5000	0.5000	0.5000	0.03618 (15)	
N1	0.6201 (3)	0.32862 (19)	0.4879 (2)	0.0421 (5)	
C1	0.5276 (4)	0.2407 (3)	0.4281 (3)	0.0526 (7)	
H1	0.4095	0.2555	0.3908	0.063*	
C2	0.5994 (6)	0.1285 (3)	0.4191 (4)	0.0678 (9)	
H2	0.5305	0.0693	0.3770	0.081*	
C3	0.7731 (6)	0.1062 (3)	0.4729 (4)	0.0735 (10)	
H3	0.8250	0.0317	0.4676	0.088*	
C4	0.8695 (4)	0.1951 (3)	0.5348 (4)	0.0629 (8)	
H4	0.9881	0.1819	0.5722	0.076*	
C5	0.7896 (4)	0.3040 (3)	0.5412 (3)	0.0486 (6)	
H5	0.8560	0.3636	0.5844	0.058*	
N2	1.0498 (3)	0.6620 (2)	0.7423 (2)	0.0501 (5)	
O1	1.0070 (3)	0.5655 (2)	0.6942 (2)	0.0635 (6)	
O2	1.1995 (4)	0.6760 (3)	0.8009 (3)	0.0956 (10)	
O3	0.9347 (5)	0.7424 (3)	0.7281 (3)	0.0905 (9)	
O4	0.7425 (3)	0.5820 (2)	0.4544 (2)	0.0624 (6)	
H1O4	0.7224	0.6435	0.4154	0.094*	0.667
H2O4	0.8088	0.5431	0.4141	0.094*	0.667
H3O4	0.8104	0.5993	0.5166	0.094*	0.667
O5	0.5815 (4)	0.5041 (2)	0.6930 (2)	0.0670 (6)	
H1O5	0.5830	0.4380	0.7239	0.101*	0.667
H2O5	0.6849	0.5295	0.7147	0.101*	0.667
H3O5	0.5148	0.5457	0.7304	0.101*	0.667

### Atomic displacement parameters (Å^2^)

**Table:**

	*U*^11^	*U*^22^	*U*^33^	*U*^12^	*U*^13^	*U*^23^
Ni1	0.0330 (2)	0.0335 (2)	0.0415 (2)	−0.00085 (16)	0.00174 (15)	−0.00095 (18)
N1	0.0411 (10)	0.0356 (10)	0.0498 (11)	0.0028 (8)	0.0065 (9)	−0.0007 (9)
C1	0.0526 (15)	0.0430 (14)	0.0613 (17)	−0.0008 (12)	0.0017 (13)	−0.0071 (12)
C2	0.083 (2)	0.0423 (16)	0.079 (2)	−0.0013 (15)	0.0110 (18)	−0.0126 (15)
C3	0.080 (2)	0.0451 (17)	0.099 (3)	0.0188 (16)	0.025 (2)	0.0026 (18)
C4	0.0477 (16)	0.0579 (18)	0.085 (2)	0.0131 (14)	0.0137 (15)	0.0159 (17)
C5	0.0403 (13)	0.0465 (14)	0.0595 (16)	0.0008 (11)	0.0069 (11)	0.0062 (12)
N2	0.0541 (13)	0.0526 (14)	0.0443 (11)	−0.0080 (11)	0.0088 (10)	−0.0055 (10)
O1	0.0690 (14)	0.0510 (13)	0.0687 (14)	−0.0078 (10)	−0.0002 (11)	−0.0095 (11)
O2	0.0725 (18)	0.123 (3)	0.0858 (19)	−0.0316 (17)	−0.0137 (15)	−0.0169 (18)
O3	0.104 (2)	0.0689 (17)	0.102 (2)	0.0254 (16)	0.0278 (18)	−0.0123 (15)
O4	0.0518 (12)	0.0568 (13)	0.0788 (15)	−0.0035 (10)	0.0088 (10)	0.0059 (11)
O5	0.0763 (16)	0.0655 (15)	0.0575 (13)	−0.0031 (11)	0.0000 (11)	−0.0016 (11)

### Geometric parameters (Å, °)

**Table:**

Ni1—O4	2.113 (2)	C4—H4	0.9300
Ni1—O5	2.128 (2)	C5—H5	0.9300
Ni1—N1	2.140 (2)	N2—O2	1.216 (4)
N1—C1	1.333 (4)	N2—O1	1.238 (3)
N1—C5	1.340 (3)	N2—O3	1.238 (4)
C1—C2	1.381 (4)	O4—H1O4	0.8200
C1—H1	0.9300	O4—H2O4	0.8200
C2—C3	1.365 (5)	O4—H3O4	0.8200
C2—H2	0.9300	O5—H1O5	0.8200
C3—C4	1.364 (5)	O5—H2O5	0.8200
C3—H3	0.9300	O5—H3O5	0.8200
C4—C5	1.369 (4)		
			
O4—Ni1—O4^i^	180.00 (11)	C4—C3—C2	118.9 (3)
O4—Ni1—O5^i^	85.71 (10)	C4—C3—H3	120.6
O4^i^—Ni1—O5^i^	94.29 (10)	C2—C3—H3	120.6
O4—Ni1—O5	94.29 (10)	C3—C4—C5	119.3 (3)
O4^i^—Ni1—O5	85.71 (10)	C3—C4—H4	120.4
O5^i^—Ni1—O5	180.000 (1)	C5—C4—H4	120.4
O4—Ni1—N1	91.23 (9)	N1—C5—C4	123.1 (3)
O4^i^—Ni1—N1	88.77 (9)	N1—C5—H5	118.5
O5^i^—Ni1—N1	89.46 (9)	C4—C5—H5	118.5
O5—Ni1—N1	90.54 (9)	O2—N2—O1	120.5 (3)
O4—Ni1—N1^i^	88.77 (9)	O2—N2—O3	122.1 (3)
O4^i^—Ni1—N1^i^	91.23 (9)	O1—N2—O3	117.3 (3)
O5^i^—Ni1—N1^i^	90.54 (9)	Ni1—O4—H1O4	112.9
O5—Ni1—N1^i^	89.46 (9)	Ni1—O4—H2O4	117.0
N1—Ni1—N1^i^	180.000 (1)	H1O4—O4—H2O4	105.0
C1—N1—C5	116.9 (2)	Ni1—O4—H3O4	110.9
C1—N1—Ni1	121.15 (19)	H1O4—O4—H3O4	106.6
C5—N1—Ni1	121.94 (19)	H2O4—O4—H3O4	103.5
N1—C1—C2	123.0 (3)	Ni1—O5—H1O5	112.2
N1—C1—H1	118.5	Ni1—O5—H2O5	116.2
C2—C1—H1	118.5	H1O5—O5—H2O5	103.3
C3—C2—C1	118.9 (3)	Ni1—O5—H3O5	113.0
C3—C2—H2	120.6	H1O5—O5—H3O5	107.4
C1—C2—H2	120.6	H2O5—O5—H3O5	103.7

Symmetry codes: (i) −*x*+1, −*y*+1, −*z*+1.

### Hydrogen-bond geometry (Å, °)

**Table:**

*D*—H···*A*	*D*—H	H···*A*	*D*···*A*	*D*—H···*A*
O4—H1O4···O2^ii^	0.82	2.39	3.209 (4)	174
O4—H2O4···O1^iii^	0.82	2.26	3.077 (4)	179
O4—H3O4···O1	0.82	2.32	3.087 (3)	157
O5—H1O5···O3^iv^	0.82	2.28	3.091 (4)	169
O5—H2O5···O1	0.82	2.43	3.191 (4)	155
C2—H2···O1^v^	0.93	2.50	3.310 (4)	145
C4—H4···O2^vi^	0.93	2.54	3.461 (4)	170

Symmetry codes: (ii) *x*−1/2, −*y*+3/2, *z*−1/2; (iii) −*x*+2, −*y*+1, −*z*+1; (iv) −*x*+3/2, *y*−1/2, −*z*+3/2; (v) *x*−1/2, −*y*+1/2, *z*−1/2; (vi) −*x*+5/2, *y*−1/2, −*z*+3/2.
